# Calcification and abscess formation around the catheter tip of a central venous access port: a case report

**DOI:** 10.1186/s13256-019-2333-z

**Published:** 2020-01-16

**Authors:** Tomoya Takami, Keisuke Fukuda, Koji Yasuda, Nozomi Kasyu, Hiroyuki Yoshitake, Kotaro Hatano, Naoki Kataoka, Tomoyuki Yamaguchi, Masafumi Tomita, Yoshiharu Shono, Shinichiro Makimoto

**Affiliations:** Department of General Surgery, Cardiology, Kishiwada Tokushukai Hospital, 4-27-1 Kamoricho, Kishiwada-shi, Osaka-fu 596-0042 Japan

**Keywords:** Central venous catheter, CVC complications, Vascular calcification, Deep neck abscess

## Abstract

**Background:**

Thrombosis of the internal jugular vein occasionally occurs in association with long-term placement of a central venous catheter; however, such complications rarely involve calcification within the blood vessels. We report a case of calcification and abscess formation around a central venous catheter tip.

**Case presentation:**

Our patient was an 84-year-old Asian woman who developed a fever that had started approximately 5 months after the placement of a central venous catheter. At the time of presentation, blood tests showed a marked inflammatory response, and chest computed tomography showed a high absorption area and air density around the catheter tip. Therefore, the patient was diagnosed with abnormal intravascular calcification and a deep neck abscess associated with long-term central venous catheter placement. The initial plan was to administer antibiotics and remove the central venous catheter. However, central venous catheter removal was deemed difficult due to the calcification and therefore required an incision. Because of the patient’s advanced age and dementia, her family requested antibiotic treatment only. Following antibiotic treatment, the patient’s inflammatory response normalized, and her fever resolved. The treatment was discontinued, and the patient’s condition gradually stabilized.

**Conclusions:**

Catheter-related complications of central venous catheter placement include vascular occlusion, extravasation of the infusion, and infection. However, abnormal calcification in the blood vessels is extremely rare, and there has been only one case report of a neonate with central venous catheter-related vascular calcification in Japan. The etiology of intravascular calcification is considered to be related to the infusion content and the infusion rate of high caloric infusions and blood products. The incidence of complications associated with long-term central venous catheter placement is expected to increase with the increasing aging of the population and advances in chemotherapy. The report of the clinical course of this rare case adds to the body of knowledge in this area.

## Introduction

Thrombosis of the internal jugular vein is a relatively rare form of deep vein thrombosis, but this condition has been reported in association with long-term placement of a central venous catheter (CVC) [1, 2]. However, it is extremely rare for such complications to involve calcification in the blood vessels [3]. We report a case in which a long-term indwelling CVC caused a deep neck abscess and was difficult to remove due to calcification around the catheter tip.

## Case presentation

Our patient was an 84-year-old Asian woman who had experienced persistent anorexia since being treated for acute myocardial infarction about 1 year prior to the current presentation. Her anorexia was thought to be related to aging. Because it was difficult to secure a peripheral venous infusion route, a CVC was placed about 5 months before the current presentation to ensure the provision of adequate nutrition. After CVC placement, the patient’s condition remained stable, but she developed a persistent fever. Infection around the CVC was suspected, so she was referred to our hospital. Her medical history included hypertension and dementia. Her regular medications were antiplatelet drugs, proton pump inhibitors, laxatives, and diuretics.

**Fig. 1:**
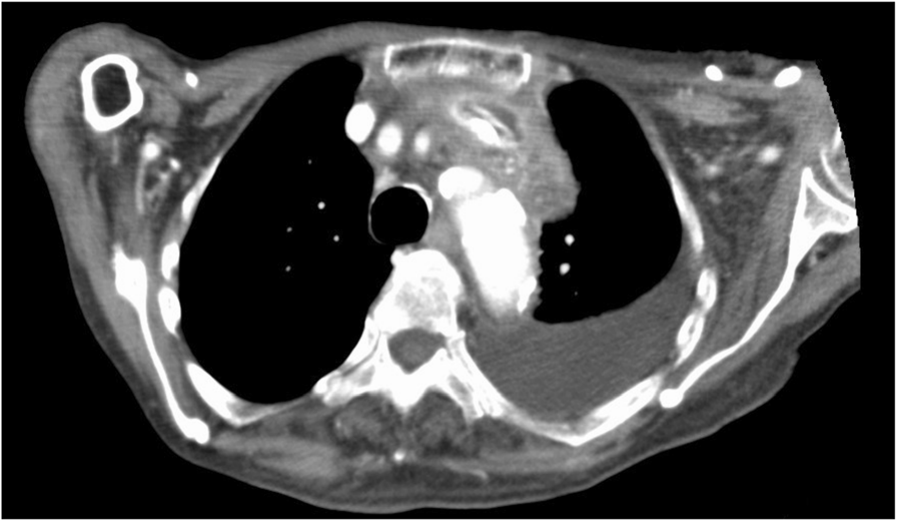
Chest computed tomography scan (axial). The catheter tip was located within the lumen of the left brachiocephalic vein, but there was a high absorption area around it with some air density

**Fig. 2:**
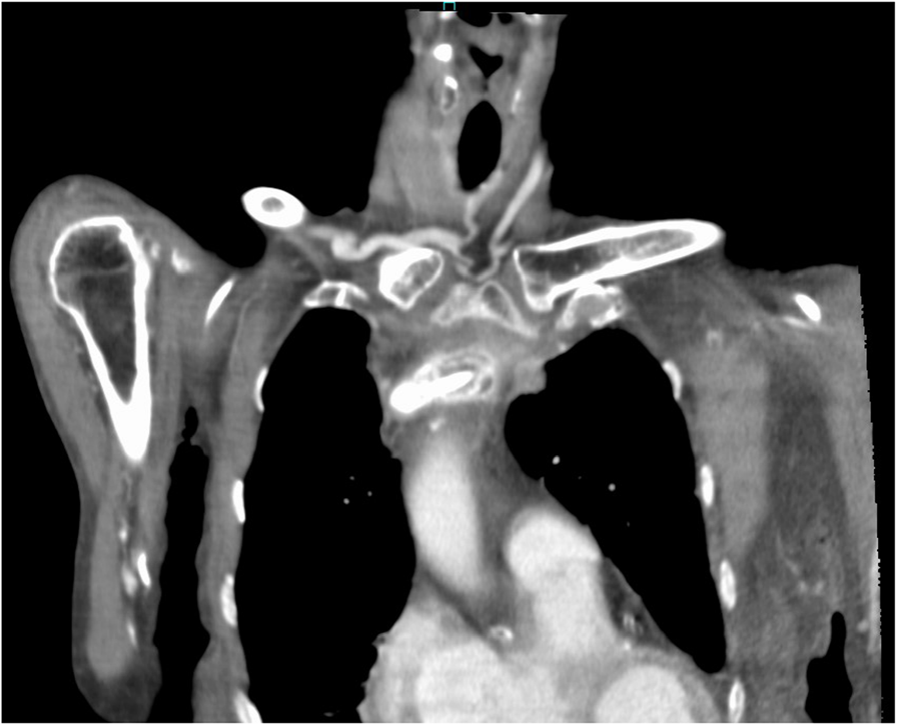
Chest computed tomography scan (coronal)

**Fig. 3:**
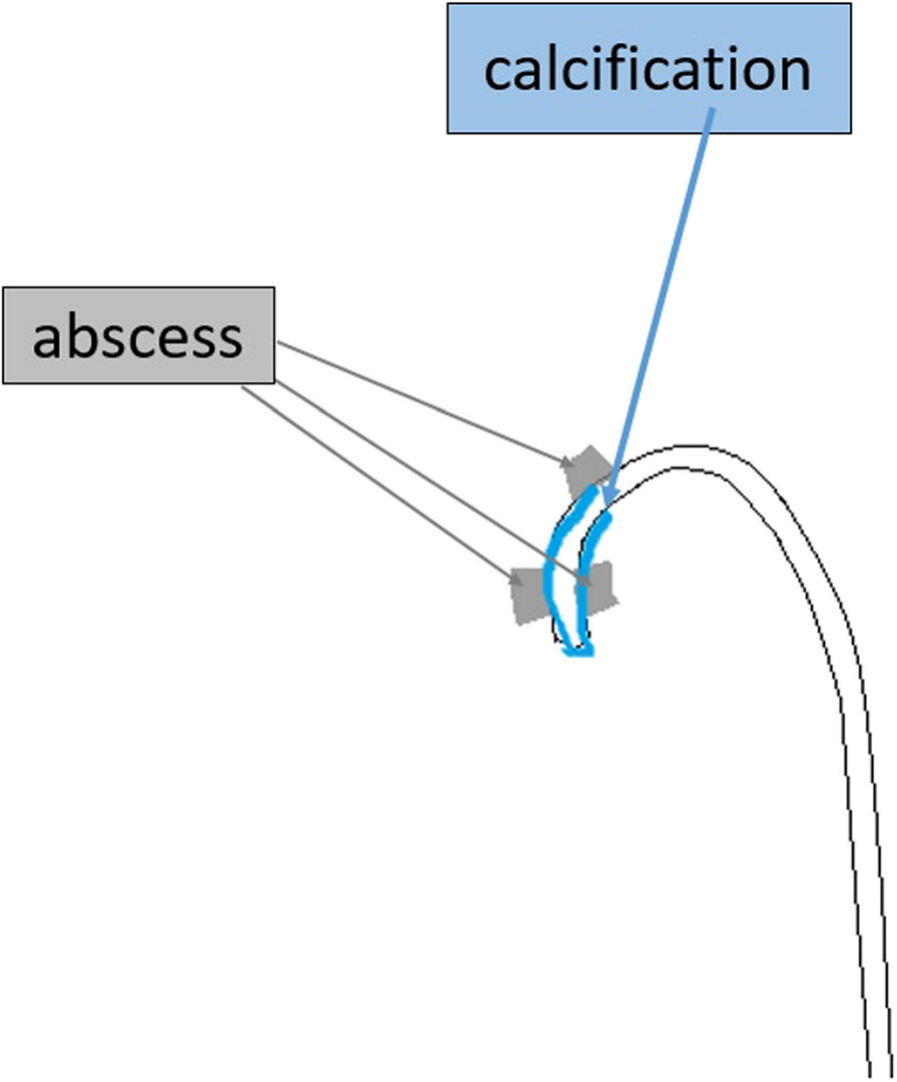
Chest computed tomography scan (sketch)

On physical examination, the patient had a temperature of 37 °C, blood pressure of 142/91 mmHg, heart rate of 93 beats/minute, and respiratory rate of 18 breaths/minute. No redness or warmth was noted around the CVC port. Blood tests revealed a marked inflammatory response. The patient’s white blood cell count was 16,600/μl, C-reactive protein concentration was 9.42 mg/dl, hemoglobin concentration was 11.2 g/dl, platelet count was 139,000/μl, blood urea nitrogen concentration was 23 mg/dl, and creatinine concentration was 0.69 mg/dl. Chest computed tomography (CT) showed a CVC port located subcutaneously in the left anterior chest, but there were no signs of infection such as a subcutaneous abscess around the port or increased fat deposition. The catheter tip was located within the lumen of the left brachiocephalic vein, but there was a high absorption area around it with some air density (Figs. 1, 2, and 3); thus, venous wall calcification and abscess formation were suspected. Chest CT showed bilateral pleural effusion that was worse on the left. Based on the blood test and CT findings, the diagnosis was abscess formation with venous wall calcification following long-term CVC placement.

The patient was hospitalized and started on antibiotics (cefmetazole 1 g every 8 hours). Bacterial culture of a blood sample collected on the day of hospitalization revealed multidrug-resistant *Serratia marcescens*, so the antibiotic was changed to meropenem (1 g every 8 hours) for a total of 15 days. The patient’s clinical course was uneventful, her fever subsided, and the inflammatory response improved. Chest CT performed 20 days after hospital admission showed that the abscess cavity had shrunk and that there was no infection recurrence. The patient was subsequently followed up at our hospital for about 1 month.

In general, cases of catheter-associated infection warrant early removal of the catheter. However, in the present case, the catheter tip was fixed to the calcified venous wall, and forcible removal would have damaged the catheter and left parts of it in the blood vessel. Therefore, surgical removal was recommended, but the family declined surgery because of the patient’s advanced age, so only antibiotic treatment was administered. Although the patient’s family was advised that the infection could recur around the CVC, they elected to avoid surgical CVC removal for the same reason.

## Discussion

Cases of infection and deep vein thrombosis associated with CVC placement have been reported [4, 5], but intravascular calcification is a rare complication [6]. To our knowledge, this is the first report of calcification and abscess formation around a CVC.

Calcification around the CVC is reportedly caused by the infusion of high-calorie solutions that have a higher concentration of calcium phosphate than the contents of the central vein, which can cause rapid calcium salt precipitation in the catheter [7]. Therefore, in the present case, the vascular environment may have caused calcium salts to precipitate because the high-calorie infusion was first administered through the central vein. In addition, there have been reports of central vein calcification due to the long-term mechanical stress associated with CVC insertion [8] and due to thrombus formation [9, 10]. The etiology of thrombus formation is associated with Virchow’s triad, which comprises endothelial injury/dysfunction (vascular wall factors), hemodynamic changes (blood flow factors), and hypercoagulability (blood characteristic factors) [11]. These three factors are thought to act alone or in combination to cause thrombus formation. Furthermore, because the CVC in the present case had been left *in situ* for a long time, the vascular endothelial cells had been physically damaged, the blood flow dynamics had been altered due to the catheter placement, and there were coagulation abnormalities due to the infection; these factors all contributed to the venous calcification.

In general, an infected CVC should be removed. However, calcification can damage the catheter tip, leaving some remnant fragments in the blood vessel after the catheter is “successfully” withdrawn. This situation will eventually require surgical resection, but only conservative treatment was administered in our patient’s case, because the her family did not want her to undergo surgery due to her advanced age.

Surgical resection is also generally recommended for blood clots around CVCs, but long-term indwelling CVCs tend to cause calcification and abscess formation. Therefore, CVCs should be removed as soon as possible after treatment is discontinued [4, 12, 13]. Although it is desirable to remove the CVC as soon as its function is complete, the appropriate timing of CVC removal may be difficult to gauge in Japan, because common alternatives to oral ingestion include a gastric fistula, feeding tube, and infusion.

In our patient’s case, the CVC was placed because the facility where the patient was being treated was unable to manage the feeding tube. In Japan, it is common for gastric fistulas or infusions to be initiated to treat physical deterioration due to poor food intake. Furthermore, in Japan, when a patient is incapable of making their own medical decisions, nutritional tubes are often selected in accordance with the wishes of the patient’s family, and a CVC is selected by the facility or hospital. However, long-term use of equipment for parenteral nutrition causes complications such as infection. Therefore, to prevent complications such as the infection and venous calcification seen in our patient’s case, it is necessary to reexamine the indications for an indwelling CVC for parenteral nutrition. In recent years, an increasing number of people and facilities in Japan have chosen not to use a gastric fistula or infusion to manage patients with poor food intake due to aging. However, this issue involves complex ethical considerations, because many families still desire parenteral nutrition for their older adult relatives; this problem needs to be discussed throughout Japan.

## Conclusions

Long-term CVC placement may result in various complications, but this is the first report of calcification with abscess formation. In our patient’s case, improvement was achieved with antibiotic treatment alone, which was selected because surgical treatment of the deep neck abscess was considered too invasive due to her advanced age. The CVC was removed as soon as it had served its purpose; however, our patient’s case highlights the need to reconsider the indications for CVC placement.

## Data Availability

Not applicable.

## Abbreviations

CT: Computed tomography
CVC: Central venous catheter
